# High-altitude exposure and ischemic stroke: pathophysiological mechanisms and current perspectives

**DOI:** 10.3389/fneur.2026.1830751

**Published:** 2026-07-20

**Authors:** Yuhuan Qiao, Yuding Luo, Yu Hu, Chuanxi Duan, Junhao Li, Xiaojing Luo, Jian Wang

**Affiliations:** 1 Department of Neurology, The Affiliated Hospital, Southwest Medical University, Luzhou, China; 2 Department of Neurology, Ya'an People's Hospital, Ya'an, China; 3 North Sichuan Medical College, Nanchong, China

**Keywords:** acclimatization, high altitude, hypoxia, inflammation, ischemic stroke, oxidative stress

## Abstract

Stroke remains the second leading cause of death worldwide and the third leading cause of disability-adjusted life year lost. In recent years, environmental and geographic determinants have been increasingly recognized as key contributors to stroke risk. High-altitude environments—characterized by chronic hypoxia, hypobaria, and elevated ultraviolet radiation—exert profound effects on cardiovascular and cerebrovascular physiology and substantially elevate stroke burden. A comprehensive understanding of how high altitude modulates the pathophysiology of ischemic stroke is therefore critical to optimizing prevention and tailored management in high-altitude populations.

This narrative review synthesizes current evidence from peer-reviewed, English-language studies identified primarily through PubMed and supplemented by Google Scholar up to December 2025. We focus on three interrelated domains: physiological adaptation, maladaptive injury, and their implications on ischemic stroke under high-altitude conditions. Under mild-to-moderate hypoxic exposure, the human body achieves acclimatization via coordinated compensatory responses, including hematologic remodeling, enhanced ventilatory function, regulation of cerebral blood flow, and adaptive cardiac remodeling. With progressive increases in altitude or prolonged hypoxic exposure, however, these compensatory mechanisms become inadequate and shift toward maladaptation. This maladaptive transition is characterized by excessive erythropoiesis, heightened blood viscosity, hypercoagulability, endothelial dysfunction, blood-brain barrier disruption, amplified neuroinflammation, and oxidative stress. Collectively, these pathological cascades promote thrombogenesis and neuronal injury, thereby increasing susceptibility to ischemic stroke at high altitude.

Future research priorities include the clarification of mechanisms governing the transition from physiological acclimatization to maladaptive injury, the identification of factors influencing individual responses to hypoxic exposure, the evaluation of targeted interventions capable of preserving beneficial adaptation or attenuating pathological processes, and the establishment of evidence-based prevention and management strategies for ischemic stroke in high-altitude populations.

## 1 Introduction

Stroke remains a leading cause of mortality and long-term disability worldwide, imposing a substantial burden on public health systems (1). It is currently the second leading cause of death and the third leading contributor to disability-adjusted life years (DALYs) lost globally (2). Notably, the stroke burden exhibits marked regional heterogeneity, with considerable variation in risk factor profiles across populations. Although stroke representss a universal health challenge, a disproportionate share of this burden falls on low- and middle-income countries, which account for approximately 87% of stroke-related deaths and 89% of DALYs lost worldwide (3). Ischemic stroke constitutes the predominant subtype, comprising roughly 65% of all incident strokes globally (3), serving as the principal driver of the worldwide cerebrovascular disease burden.

In recent years, environmental and geographic determinants have garnered increasing attention as important modifiers of stroke risk. High-altitude environments are characterized by a constellation of environmental stressors, including reduced barometric pressure, diminished oxygen availability, low ambient temperatures, and increased ultraviolet radiation. Among these, hypobaric hypoxia is regarded as the primary stimulus driving physiological adaptation and high-altitude-related disorders (4). Additional factors—such as dehydration (5), cold exposure (5), oxidative stress (6), and secondary erythrocytosis (7)—may further contribute to stroke pathogenesis under high-altitude conditions (8). According to widely used classifications, altitude is categorized as high altitude (1,500–3,500 m), very high altitude at (3,500–5,500 m), and extreme altitude (>5,500 m) (9, 10).

The reduction in ambient oxygen partial pressure at high altitude elicits a cascade of physiological responses, including hyperventilation (11, 12), increased cardiac output (13), and augmented erythropoiesis (7). During the initial phase of exposure, these responses serve to maintain oxygen delivery and tissue perfusion and therefore represent critical compensatory mechanisms. However, with prolonged or sustained exposure, these responses may progressively destabilize physiological homeostasis and impose chronic pathological stress on the cardiovascular and cerebrovascular systems (11, 14). High-altitude exposure not only increases the risk of altitude-related illnesses and hypoxic brain injury but may also unmask previously subclinical cerebral lesions under hypoxic or hypobaric conditions, manifesting as focal neurological deficits (15, 16), seizures (16, 17), or cognitive impairment (18, 19).

As estimated 500 million people reside at elevations exceeding 1,500 m (20), many in regions with limited healthcare resources. Consequently, stroke in high-altitude settings has emerged as an important focus of neuroepidemiological research (21). Epidemiological findings to date, however, remain heterogeneous. Several studies have reported lower stroke mortality or reduced hospital admission rates at intermediate altitudes (approximately 1,500–3,500 m) (22–25), whereas others have documented increased stroke incidence, earlier onset, and greater clinical severity at very high altitudes (> 3,500 m) (26–31). These observations suggest that the relationship between high-altitude exposure and stroke risk is unlikely to be linear or simply dose dependent (24). Importantly, these seemingly discordant findings may partly reflect methodological differences in study endpoints and population characteristics. Some investigations assessed stroke mortality or prevalence among long-term high-altitude residents, while others focused on incidence, clinical severity, age at onset, or neuroimaging features in hospitalized patients. Furthermore, study populations have ranged from indigenous high-altitude dwellers to lowland migrants and military personnel undergoing variable durations of high-altitude exposure. Such heterogeneity in design and sampling likely contributes to the inconsistent epidemiological landscape. Representative studies examining high-altitude exposure and stroke outcomes are summarized in Table 1.

**Table 1:** Articles related to high-altitude exposure and stroke.

Author (Year)	Article type	Study population	Altitude	Outcome
Feah et al. (22)	Clinical study	Nationals born in Switzerland	259–1,960 m	The mortality risk for stroke decreased after adjustment by 12% with an increase of 1,000 m in the altitude of the place of reference
Ortiz-Prado et al. (23)	Review	An ecological analysis in Ecuador from 2001–2017.	<1,500 m 1,500–2,500 m 2,500–3,500 m 3,500–5,500 m	Lower stroke mortality and hospital admission at 2,000–3,500 m
Hameed et al. (24)	Review	Studies about ischemic stroke at high altitudes	2,500–5,800 m	high altitudes of >3,500 m increase the risk of ischemic stroke but when people reside between 1,500 to 2,500 m, there appears to be a protective effect for stroke
Ortiz-Prado et al. (25)	Review	17 documents	1,500–3,500 m >3,500 m	a window around 2,000–3,500 m of elevation might be enough to generate some protective mechanisms
Jaillard et al. (26)	Clinical study	Individuals over 15 years old	Cuzco (3,380 m)	High altitude was associated with higher stroke prevalence.
Jha et al. (27)	Clinical study	Indian soldiers	>4,270 m	Stroke admission rate 13.7/1,000 vs. 1.05/1,000 at low altitude
Liu et al. (28)	Clinical study	Cases of first-ever acute ischemic strokes	Penglai (20 m) Huzhu (2,550 m) Yushu (4,200 m)	Earlier onset and larger infarct volume at high altitude
Yan et al. (29)	Clinical study	Patients with first-episode acute ischemic stroke	Tianjin (3.5 m) Xining (2,275 m)	Patients at high altitudes showed a significant trend toward lower age, larger infarct volume and worse prognosis
Lu et al. (30)	Clinical study	Patients with ischemic stroke	Tibet (3,650 m) Beijing (40 m)	Young adult stroke was more predominant in Tibet
Zheng et al. (31)	Review	17 studies from four continents	1,500 m to nearly 5,000 m	The stroke prevalence we observed in high-altitude areas exceeded the world average

Evidence indicates that moderate hypoxic exposure at intermediate altitudes may enhance cerebrovascular autoregulation and promote metabolic adaptation (32–34). In contrast, exposure to very high altitude may precipitate excessive erythrocytosis, increased blood viscosity (7), endothelial dysfunction (35, 36), and inflammatory activation (37, 38), thereby amplifying stroke risk (31). These effects also appear to differs across populations. Indigenous high-altitude groups, such as Tibetans, maintain higher oxygen saturation together with lower hemoglobin and hematocrit levels compared with lowland migrants, reflecting more efficient adaptation to chronic hypoxia (39–42). Conversely, lowland migrants often exhibit less efficient physiological acclimatization and greater susceptibility to maladaptive responses—particularly excessive erythrocytosis—during prolonged high-altitude residence (43).

**Figure 1:**
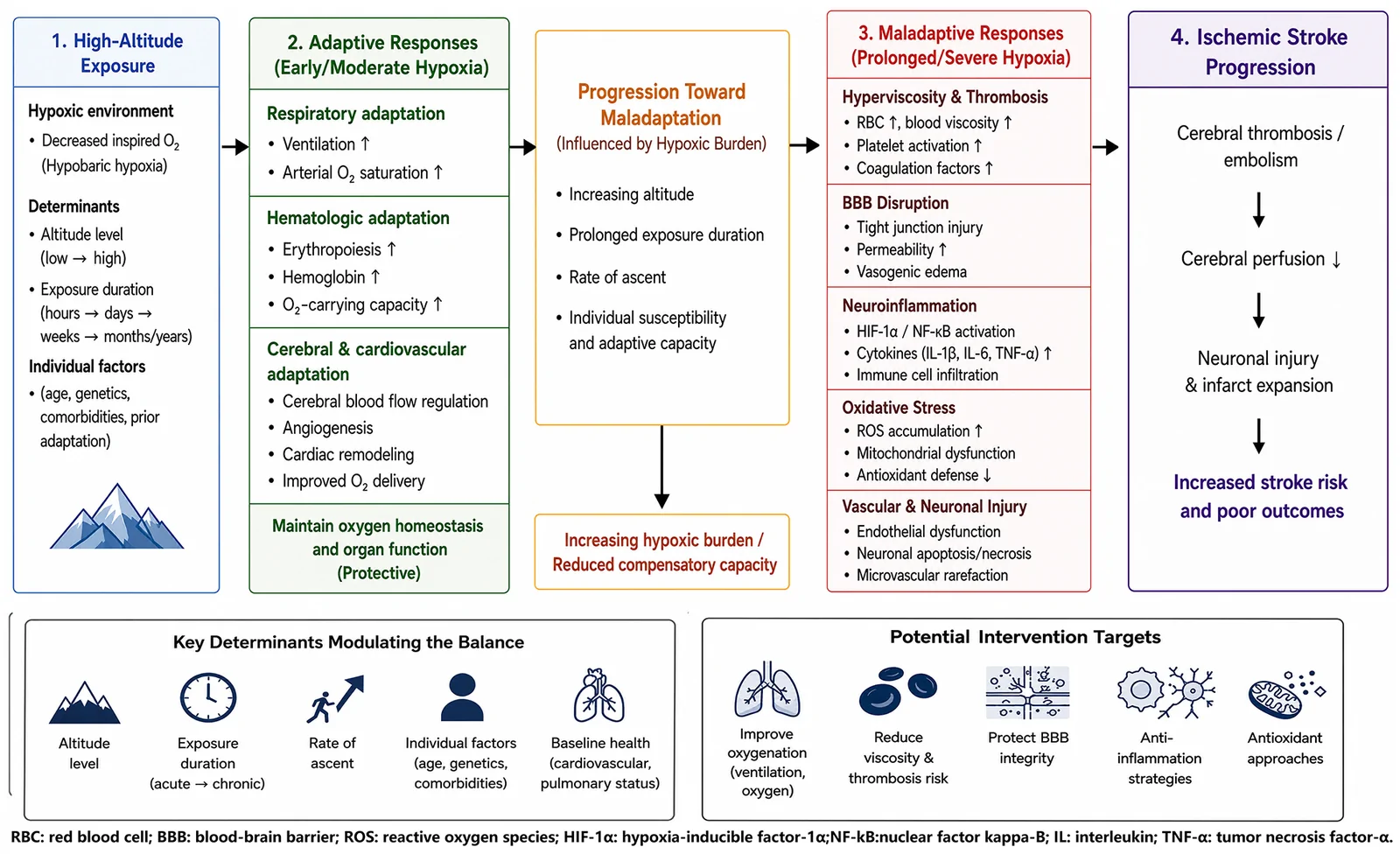
Adaptive and maladaptive mechanisms linking high-altitude exposure and ischemic stroke.

Given the complexity and heterogeneity of these interactions, an integrated mechanistic framework is required to elucidate the relationship between high-altitude exposure and ischemic stroke. Unlike prior reviews that predominantly emphasized epidemiological associations or isolated pathological mechanisms, the present review adopts a dynamic adaptation–maladaptation framework. Within this model, high-altitude exposure induces a biphasic response characterized by early compensatory acclimatization followed by progressive maladaptive injury under prolonged or extreme hypoxic conditions. Specifically, this review synthesizes altitude-dependent physiological responses, exposure duration, hematologic remodeling, cerebral blood flow (CBF) regulation, endothelial dysfunction, blood-brain barrier (BBB) disruption, neuroinflammation, oxidative stress, and thrombogenesis into a unified conceptual model. The major mechanisms potentially linking high-altitude exposure to ischemic stroke are illustrated in Figure 1.

A more nuanced understanding of these mechanisms may clarify the complex, non-linear association between altitude and ischemic stroke and provide a theoretical foundation for altitude-specific prevention strategies and tailored clinical interventions in high-altitude populations.

Acute to moderate hypoxic exposure elicits coordinated adaptive responses, including ventilatory compensation (11, 12, 44), hematologic remodeling (45–47), tight regulation of cerebral blood flow (32, 33, 48), and cardiovascular compensation (49, 50). With progressive increases in altitude or prolonged hypoxic exposure, excessive hypoxic stress may promote maladaptive changes, such as excessive erythrocytosis (8, 51–54), blood-brain barrier disruption (55), inflammatory activation (56, 57), and oxidative stress (6, 58–60), thereby increasing susceptibility to ischemic stroke. This figure provides an interpretative synthesis of currently evidence and illustrates a conceptual continuum from acclimatization to maladaptation, rather than prescribing specific altitude- or time-dependent physiological threshold.

## 2 Literature search strategy

This narrative review was conducted by systematically searching PubMed and Google Scholar for English-language studies examining the relationship between high-altitude exposure and ischemic stroke. The literature search included publications available up to December 31, 2025. Search strings combined keywords and Medical Subject Headings terms related to “high altitude,” “plateau,” “hypoxia,” “ischemic stroke,” “acute ischemic stroke,” “pathophysiology,” “mechanism,” “cerebral blood flow,” “erythrocytosis,” “blood-brain barrier,” “inflammation,” and “oxidative stress.”

Studies addressing high-altitude-related physiological adaptation, cerebrovascular regulation, ischemic stroke mechanisms, epidemiology, and clinical management were prioritized for inclusion. Additional relevant references were identified through manual screening of bibliographies from eligible articles. Non-English publications, conference abstracts without accessible full text, and studies lacking direct relevance to cerebrovascular or hypoxia-related mechanisms were excluded. The final selection of literature was guided by its relevance to the central theme of adaptive and maladaptive responses to high-altitude exposure in the context of ischemic stroke.

As a narrative review, this article does not adhere to a formal systematic review protocol or reporting guideline. Nevertheless, the literature search and study selection process were informed by published methodological recommendations for narrative reviews (61).

## 3 Acclimatization and protective responses under high-altitude exposure

High altitude exposure is not uniformly pathogenic; indeed, acclimatization and endogenous protective responses are frequently observed at moderate elevations (23–25). Mild-to-moderate hypoxia can promote tissue-level functional compensation (62) through enhanced cerebrovascular autoregulation (32–34), metabolic adaptation (62, 63), and preservation of neurovascular unit integrity (64), collectively mitigating susceptibility to ischemic brain injury (62, 65).

### 3.1 Ventilatory compensation

One of the earliest and most fundamental adaptive responses to high-altitude ascent is sustained augmentation of ventilatory function, characterized by increased respiratory rate, greater tidal volume, and a marked elevation in minute ventilation (11, 12, 44). This response is triggered by reductions in inspired oxygen partial pressure and arterial oxygen tension (PaO_2_), which activate peripheral chemoreceptors–principally the carotid bodies–initiating the hypoxic ventilatory response (66).

Although hyperventilation improves arterial oxygenation, it simultaneously reduces arterial carbon dioxide tension (PaCO_2_), producing hypocapnia and consequent respiratory alkalosis. During acclimatization, end-tidal CO_2_ progressively declines (67). Over days to weeks, renal compensation develops through increased bicarbonate excretion, generating a relative metabolic acidosis that partially offsets the alkaline shift and restores arterial pH toward baseline (68, 69). This pH normalization sustains ventilatory drive by counteracting the inhibitory influence of hypocapnia on central respiratory centers (68).

The time required to achieve ventilatory compensation is altitude dependent, typically requiring approximately 4 days at 3,000 m, 8 days at 4,000 m, at least 2 weeks at 5,000 m, and 4–6 weeks above 6,000 m (70). Nevertheless, even after complete acclimatization, ventilation can only partially correct hypoxemia and cannot restore PaO_2_ to sea-level values (13, 44, 66). Sustained ventilatory enhancement therefore constitutes a cornerstone of physiological acclimatization to hypoxic environments.

Given that CBF is tightly coupled to PaO_2_ and PaCO_2_, ventilatory adaptation exerts a critical influence on cerebral perfusion under hypoxic conditions (32, 48, 70). Hypoxia, hypotension, and aberrant CO_2_ levels can reduce CBF and cerebral perfusion pressure (32, 48, 70), thereby exacerbating cerebral ischemic injury (71). In severe cases, altered mental status (72), cerebral edema (73), or brainstem involvement (74, 75) may precipitate abnormal respiratory patterns or respiratory failure (76), further aggravating cerebral ischemia (71, 74). Thus, sustained ventilatory augmentation during high-altitude exposure helps preserve systemic oxygenation and attenuates early hypoxemic stress. Whether these physiological adaptations translate into measurable improvements ischemic stroke outcomes, however, remains uncertain.

### 3.2 Hematologic remodeling

A hallmark physiological response to high-altitude exposure in lowland individuals is hematologic remodeling, which constitutes a central component of acclimatization aimed at preserving tissue oxygen delivery under hypoxic conditions (47).

During the initial phase of ascent, hypobaric hypoxia and concomitant dehydration induce hemoconcentration, rapidly elevating hemoglobin concentration and augmenting arterial oxygen content. Concurrently, increased intracellular 2,3-diphosphoglycerate reduces hemoglobin-oxygen affinity and shifts the oxygen-hemoglobin dissociation curve to the right, thereby facilitating oxygen unloading to peripheral tissues (45, 46).

With continued exposure, transient hemoconcentration is progressively supplanted by hypoxia-driven erythropoiesis. Hypoxia-inducible factors (HIF) stabilize under low oxygen tension and upregulate renal erythropoietin (EPO) secretion (77, 78), stimulating bone marrow erythropoiesis and producing moderate, sustained increases in erythrocyte mass and hemoglobin concentration. Renal EPO production approximately doubles at 3,000 m and triples at 4,000 m (77–79). Upon descent to low altitude, EPO levels decline rapidly, and hematologic parameters normalize, underscoring the reversibility of this adaptive response (80–82).

From a cerebrovascular perspective, elevated red blood cell distribution width has been independently associated with increased stroke risk, greater infarct severity, and poorer post-stroke functional outcomes (83). In contrast, moderate increases in hemoglobin concentration during early hypoxic exposure may enhance arterial oxygen content and facilitate tissue oxygen delivery in the setting of compromised cerebral perfusion (84, 85). Given that acute ischemic stroke is characterized by arterial occlusion, reduced cerebral perfusion, and resultant metabolic and neuronal injury (1), early-phase hematologic adaptations may theoretically attenuate hypoxic cerebral injury. Nevertheless, whether these physiological adjustments translate into meaningful protection against ischemic injury or improved clinical outcomes remains uncertain.

Notably, high-altitude-adapted populations exhibit hematologic profiles that differ fundamentally from those of lowland sojourners. Tibetans highlanders, for example, maintain relatively low hemoglobin concentrations while achieving efficient oxygen utilization and tissue oxygen delivery (47, 86–88) through complementary adaptive mechanisms, including enhanced ventilatory drive and microcirculatory optimization. These phenotypic pattern implies that the pronounced early erythropoietic response typical of lowland individuals represents a transitional compensatory mechanism rather than a stable, long-term evolutionary adaptation to high-altitude hypoxia.

### 3.3 Cerebral blood flow regulation

CBF is governed by the interplay between cerebral perfusion pressure and cerebrovascular resistance (89). Cerebral perfusion pressure is principally determined by systemic arterial pressure, whereas cerebrovascular resistance depends on vessel caliber and blood viscosity (89). During acute high-altitude exposure, systemic hypoxia induces cerebral vasodilation, producing a marked early increase in CBF that serves as an key compensatory mechanism for sustain cerebral oxygen delivery (32, 33, 48). Concomitantly, hypoxia-induced hyperventilation lowers PaCO_2_, generating hypocapnia. As a potent cerebral vasoconstrictor, hypocapnia partially offsets hypoxia-mediated vasodilation (90–92). Consequently, although CBF declines modestly from its initial peak, it remains elevated above sea-level baselines throughout the early exposure period (91, 93). Given that ischemic stroke is defined by abrupt reduction of regional CBF and resultant cerebral hypoxia (94, 95), the early hyperemic response at high altitude may represent a physiological attempt to preserve cerebral oxygenation under hypoxic conditions (91). Whether this adaptive hemodynamic response translates into measurable protection against ischemic stroke or improved clinical outcomes, however, remains uncertain.

With prolonged high-altitude exposure, plasma volume contracts (7, 96), while hemoglobin concentration and hematocrit rise (88, 97, 98), enhancing the oxygen-carrying capacity per unit of blood (84, 85). These hematologic adjustments constitute core elements of early acclimatization and contribute to the gradual rebalancing of CBF (48, 98). Over time, CBF declines toward values closer to sea-level norms, yet typically remains modestly elevated relative to lowland baselines (91, 93). This physiological trajectory reflects a transition from acute hypoxia-driven vasodilation to a more homeostatic state in which cerebral oxygen delivery is maintained despite persistently reduced ambient oxygen availability (91, 92, 99, 100).

Beyond mean flow, CBF exhibits intrinsic pulsatility modulated by cardiac cycle (101). Pulsatile dynamics are shaped by large-artery stiffness (102, 103), characteristic impedance (102), and the dampening capacity of the distal vasculature (104). Notably, as altitude increases from 1,400 m to 4,300 m, pulsatility indices in the internal carotid and middle cerebral arteries decrease progressively (105). This blunted cerebrovascular pulsatility, coupled with optimized oxygen-delivery kinetics, appears to be a distinctive hemodynamic feature of high-altitude-adapted populations and may contribute to long-term cerebral perfusion stability under chronic hypoxic conditions (106).

### 3.4 Cardiac compensation and remodeling

During the initial phase of high-altitude exposure, an increase in heart rate represents one of the earliest physiological responses (107), driven predominantly by heightened sympathetic activation (108) coupled with vagal withdrawal (109). Stroke volume remains relatively stable at rest (13); thus, the early rise in cardiac output is primarily attributable to tachycardia, which helps preserve systemic oxygen delivery despite reduced arterial oxygen content (107). Over several days of acclimatization, stroke volume gradually declines while heart rate remains elevated, allowing cardiac output to trend back toward baseline levels (49, 50). This hemodynamic evolution is thought to reflect hypoxia-induced pulmonary vasoconstriction (110, 111), contraction of plasma volume (7, 96), and altered ventricular filling dynamics (49). Importantly, global systolic and diastolic function generally remain within physiological limits throughout this transition (49, 50).

The effects of hypoxia on myocardial contractility are bidirectional, reflecting the integration of multiple neurohumoral pathways (112). Positive inotropic influences include hypoxia-driven sympathetic activation (113, 114) and engagement of the HIF-mediated apelin-APJ signaling xis (115–117). Counterbalancing mechanisms include hypoxia-stimulated nitric oxide production (118) and adenosine release (119, 120), both of which exert negative inotropic effects. In healthy individuals, these opposing pathways reach a functional equilibrium that preserves overall myocardial pump function, such that ventricular systolic performance is largely maintained (112)—and in some indices even modestly enhanced—during acute exposure (49). This resilience underscores the considerable tolerance of the normal myocardium to hypoxemic stress (50).

Cardiac performance and hemodynamic stability are intimately associated with ischemic stroke risk and may modulate cerebral perfusion under both acute and chronic conditions (121, 122). Clinically, arrhythmias (123–125), reductions in cardiac output (101, 126), and structural cardiac function (122, 127) represent major contributors to cerebrovascular events and stroke-related complications (122, 123). Moreover, acute ischemic stroke itself can provoke autonomic dysregulation and hemodynamic instability, further illustrating the bidirectional heart-brain interaction in cerebrovascular pathophysiology (122, 128, 129). In this context, early cardiac compensatory responses at high altitude may help sustain systemic hemodynamics and potentially support cerebral perfusion under hypoxic conditions. Whether these physiological adaptations translate into meaningful clinical benefits for patients with ischemic stroke, however, remains to be determined.

## 4 The transition from adaptation to maladaptation

Accumulating evidence indicates that altitude, duration of exposure, and population-specific characteristics profoundly shape physiological responses to hypobaric hypoxia (5, 40, 108, 130). As summarized in Table 2, progressive increments in elevation and exposure length are associated with stepwise alterations in ventilatory compensation (11, 12, 44), hematologic remodeling (45–47), CBF regulation (32, 33, 48), and cardiovascular acclimatization (49, 50). In parallel, severe or sustained hypoxia promotes maladaptive processes, including excessive erythrocytosis (8, 51–54), BBB disruption (55), inflammatory activation (56, 57), and oxidative stress (6, 58–60). Epidemiological and physiological data further suggest that indigenous high-altitude populations and lowland exhibit fundamentally different adaptive trajectories migrants (39–42).

**Table 2:** Representative findings related to altitude level, exposure duration, and adaptive or maladaptive mechanisms during high-altitude exposure.

Mechanism	Author (Year)	Condition	Findings
Ventilatory compensation	Jha et al. (27)	Simulated 3,000 m acute exposure (7 h)	Minute ventilation increased significantly during acute hypoxic exposure, supporting early ventilatory compensation.
	Hoiland et al. (70)	3,000 m (4 d) 4,000 m (8 d) 5,000 m (≥2 weeks) >6,000 m (4–6 weeks)	3,000 m (4 d): ventilatory acclimatization develops progressively 4,000 m (8 d): longer acclimatization required 5,000 m (≥2 weeks) and >6,000 m (4–6 weeks): prolonged exposure required for sustained ventilatory adaptation; despite acclimatization, PaO_2_ cannot return to sea-level values.
	Tominec et al. (13)	3,375 m (69 h)	Young adult stroke was more predominant in Tibet
Hematologic remodeling	Eckardt et al. (82)	3,000 m and 4,000 m (5.5 h)	EPO increased with estimated 1.8-fold increase and 3-fold increase in production rate.
	Ge et al. (80)	Simulated 1,780–2,800 m (6–24 h)	2,100–2,500 m may represent a threshold for sustained erythropoietic stimulation.
	Jaafar et al. (46)	High-altitude (4 days)	Increased erythrocyte 2, 3-DPG shifts the oxygen dissociation curve to the right and improves oxygen delivery during high-altitude adaptation.
	Mairbäurl et al. (45)	High altitude (weeks to months)	Total hemoglobin mass may increase by 20–50% depending on altitude and duration of exposure.
Cerebral blood flow regulation	Hoiland et al. (70)	3,000 m (4 days) 4,000 m (8 days) 5,000 m (≥2 weeks) >6,000 m (4–6 weeks)	3,000–4,000 m: CBF remains elevated during early acclimatization 5,000-6,000 m: prolonged acclimatization is required and CBF progressively declines toward sea-level values with increasing hematocrit and ventilatory acclimatization.
	Howe et al. (98)	5,050 m; 1 week acclimatization	Hematocrit increased from 42.5% to 49.6%, while global CBF decreased from 844 to 619 mL/min during acclimatization.
Cardiac compensation and remodeling	Naeije et al. (49)	3,800 m (8 days)	cardiac output returns toward baseline, heart rate remains increased, and stroke volume decreases.
	Williams et al. (50)	Acute hypoxia (0–12 h); Prolonged hypoxia (1 day-6 months) Lifelong hypoxia	Acute hypoxia: increased cardiac output is driven by elevated heart rate with maintained ventricular volumes. Prolonged hypoxia: haemoconcentration restores arterial oxygen content, while left ventricular filling and stroke volume decrease. Lifelong hypoxia: Sherpa show smaller LV volumes despite larger total blood volume.
	Aggarwal et al. (107)	>3,500 m for lowland men	Lowland men ascending beyond 3,500 m: increased sympathetic activity stimulates β-adrenergic receptors, increases cardiac output, and helps sustain blood pressure and peripheral oxygen delivery.
Hematologic abnormalities	Wheatley et al. (147)	8,848 m (Acute exposure) 4,559–7,549 m (Prolonged exposure)	Acute exposure initially increases hemoglobin through plasma volume reduction, whereas prolonged acclimatization increases erythropoiesis and red cell mass.
	Villafuerte et al. (88)	4,000 m (Chronic exposure) >4,300 m (Chronic exposure)	Long-term high-altitude adaptation may maintain relatively lower hemoglobin concentrations while preserving oxygen delivery and limiting hyperviscosity.
	Wang et al. (127)	3,700 m (5 month)	Long-term very high-altitude exposure impaired cardiopulmonary performance
Blood brain barrier injury	Dunn et al. (35)	Equivalent 7,900 m	Significant BBB disruption occurred only when inspired oxygen fraction declined to 0.08–0.10
Neuroinflammation and inflammatory amplification	Eltzschig and Carmeliet (182)	>3,400 m (3 days) 8,400 m (ascent)	After short-term high-altitude exposure, systemic inflammation activated under hypoxic conditions. Severe hypoxemia at extreme altitude was associated with vascular leakage and inflammatory activation.
	Liu et al. (28)	20 m, 2,550 m, and 4,200 m (Long term)	CRP levels progressively increased with altitude in acute ischemic stroke patients and were accompanied by larger infarct volumes and more frequent disturbance of consciousness at high altitude.
Oxidative stress	Pena et al. (6)	High-altitude exposure (14 days)	ROS production remained elevated from day 1 to day 14, whereas antioxidant capacity decreased during the first several days of exposure
	Mallet et al. (62)	weeks to months at altitude	Acute and long-term hypoxia increased oxidative stress,
	Zhao et al. (200)	hypoxic-ischemic brain injury	ROS rapidly increased after hypoxia-ischemia and overwhelmed antioxidant defenses.

On the basis of these observations, we propose an interpretative framework in which acclimatization and maladaptive injury represent not binary endpoints, but successive stages along a continuum of hypoxic burden. Within this model, the transition from physiological adaptation to pathological injury is unlikely to be dictated by a single altitude threshold. Instead, it emerges from the cumulative interplay of hypoxic severity, exposure duration, and individual adaptive capacity—factors that ultimately determine whether high-altitude exposure remains protective or progresses toward cerebrovascular injury and ischemic stroke.

## 5 Acclimatization failure and pathological injury under high-altitude exposure

When hypoxic stress exceeds physiological tolerance—whether due to increasing altitude, prolonged exposure, or inadequate individual acclimatization—compensatory mechanisms begin to fail and gradually give way to maladaptive pathological processes. These maladaptive transitions are characterized by increased blood viscosity (7, 131), endothelial injury (35, 36), inflammatory activation (132), and oxidative stress (58), all of which disrupt cerebrovascular homeostasis and substantially elevate susceptibility to ischemic stroke (8).

### 5.1 Hematologic abnormalities

Under conditions of prolonged exposure to very high altitude, hypoxia ceases to function primarily as an acclimatization stimulus and instead becomes a dominant pathological stressor (25, 133). A longitudinal study at approximately 3,700 m has demonstrated that otherwise healthy adults can develop maladaptive phenotypes over a 5-month exposure period, including impaired cardiopulmonary-metabolic integration (127). Among these alterations, excessive erythropoiesis and resultant hyperviscosity are especially prominent and have been strongly associated with cerebrovascular and cardiovascular complications, including ischemic stroke (8, 51–54).

Sustained hypoxic exposure drives persistent elevation of EPO and progressive erythrocytosis, producing marked increases in hemoglobin concentration and hematocrit (77–79). Beyond a critical physiological threshold, however, further expansion of erythrocyte mass no longer enhances oxygen delivery; instead, it precipitates a hyperviscous state (7, 88) that impairs pulmonary gas exchange (134, 135), alters cerebral hemodynamics (136, 137), and elevates vascular resistance (138–140). These hemorheological disturbances create a prothrombotic milieu conducive to ischemic stroke pathogenesis (131, 141–143).

Hypoxia also directly activates the coagulation cascade, as evidenced by elevated fibrinogen (130), increased D-dimer and fibrin degradation products (144, 145), enhanced platelet aggregability (145), and upregulation of coagulation factor VIII (146, 147), often in concert with endothelial injury (145, 148, 149). The concurrent expansion of circulating erythrocytes and platelets further amplifies this hypercoagulable state (150). The combined effects of hyperviscosity and coagulation activation markedly increase the risk of both arterial and venous thrombotic events, thereby reinforcing the pathological substrate for ischemic stroke at high altitude (131, 141–143).

### 5.2 Blood-brain barrier injury

The BBB is a highly specialized interface formed principally by brain microvascular endothelial cells, which segregate the systemic circulation from the central nervous system (CNS) (151). It plays a pivotal role in regulating molecular exchange, maintaining cerebral homeostasis, modulating CBF, and shielding neural tissue from potentially deleterious circulating factors (152). Under high-altitude conditions, BBB integrity becomes increasingly vulnerable to hypoxic insult (55). Notably, hypoxia-induced barrier disruption follows a threshold-dependent pattern, with significant breakdown typically occurring only when the inspiratory oxygen fraction falls to approximately 0.08 to 0.10, corresponding to an equivalent altitude of ~7,900 m (35).

Brain microvascular endothelial cells and their intercellular junctional complexes constitute the primary structural determinants of BBB integrity and are early targets of hypoxic injury. Hypoxia induces the disassembly and redistribution of tight junction proteins, thereby increase paracellular permeability (153–155). Excessive activation of matrix metalloproteinases and endothelial injury-related signaling pathways further compromise barrier function, facilitating inflammatory cell infiltration, cerebral edema, neuroinflammation, and neuronal injury (156).

Pericytes represent another critical regulator of BBB homeostasis under hypoxic conditions. Moderate hypoxia alters pericyte secretory profiles and contractile properties, influencing vascular permeability and barrier stability (157, 158). In contrast, pericyte loss or detachment destabilizes the vascular wall, exacerbating BBB disruption and microvascular leakage (159).

Astrocyte activation further contributes to barrier dysfunction. Reactive astrocytes release proinflammatory mediators and upregulate matrix metalloproteinases, promoting tight junction disruption and increasing BBB permeability (153, 160).

BBB permeability rises within hours of acute high-altitude exposure (161), with partial recovery possible through restoration of tight junction proteins and transporter function (158, 162). Nevertheless, progressive barrier impairment increases the risk of CNS dysfunction and stroke (158). Moreover, hypoxia-induced angiogenesis often yields structurally immature vessels. Although these neovessels may improve local perfusion, their inherent leakiness further undermines barrier stability (156, 163).

In the context of ischemic stroke, BBB disruption (159, 164, 165) facilitates infiltration of peripheral immune cells (166–168) and proinflammatory mediators (164, 169), amplifying tissue injury. Increased vascular permeability also permits plasma extravasation of plasma-derived fluid, resulting in vasogenic cerebral edema (165, 170–172) and exacerbating ischemic damage. Given that BBB dysfunction occurs early in stroke pathogenesis, preservation of barrier integrity has emerged as a promising therapeutic target and may correlate with improved neurological outcomes (159, 173).

### 5.3 Neuroinflammation and inflammatory amplification

Neuroinflammation plays a central role in the initiation, progression, and functional outcome of ischemic stroke, exerting both neurotoxic and reparative effects depending on its magnitude and temporal profile (56, 57). Early, well-regulated inflammatory responses facilitate clearance of necrotic debris and initiate reparative processes (174–176), whereas excessive or sustained inflammation drives secondary brain injury (132), infarct expansion (177), and neuronal death (132, 177).

Clinical studies have shown that acute ischemic stroke patients at higher altitudes exhibit progressively elevated C-reactive protein levels compared with their lowland counterparts (28). High-altitude hypoxia strongly activates HIF signaling pathways (178), particularly HIF-1α (37) and nuclear factor-κB (NF-κB) (37, 179). These transcriptional regulators orchestrate the release of proinflammatory mediators—including interleukin-6 (IL-6) (180), tumor necrosis factor-α (TNF-α) (181), and vascular endothelial growth factor (182)—which amplify neuroinflammatory cascades under hypoxic conditions (37, 38).

Key proinflammatory cytokines such as IL-1β, TNF-α, and IL-6 disrupt endothelial integrity (183) and increase BBB permeability (184), thereby promoting leukocyte adhesion (183), transmigration (185), and parenchymal infiltration (186). The resulting inflammatory amplification loop exacerbates neuronal injury and expands the penumbra (56, 132, 153). The accentuated inflammatory burden at high altitude may therefore contribute to more severe ischemic brain injury and potentially influence infarct severity and long-term neurological outcomes.

Importantly, post-stroke neuroinflammation extends beyond the infarct core, spreading through interconnected neural networks and persisting over prolonged periods (56). Necrotic neurons release damage-associated molecular patterns (DAMPs) (57), which engage pattern recognition receptors such as Toll-like receptors (57, 187) and inflammasomes (57). This innate immune activation sustains cytokine and chemokine production, perpetuating neuroinflammation and ongoing neuronal loss (57, 188). In the heightened proinflammatory milieu characteristic of high-altitude environments, these mechanisms may be further intensified, potentially exacerbating neurovascular injury (38, 189). Nonetheless, the degree to which they directly determine clinical stroke severity and long-term outcomes remains incompletely understood.

### 5.4 Oxidative stress

Oxidative stress arises from excessive accumulation of reactive oxygen species (ROS), leading to dysregulated cellular signaling, oxidative modification of biomolecules, and ultimately neuronal injury or cell death (190). Pathological ROS overproduction is a hallmark of hypoxic environments and is closely associated with ischemic brain injury (191).

Under physiological or mild stressful conditions, ROS also function as signaling molecules that activate antioxidant and anti-inflammatory pathways (4). Oxidative modification of Kelch-like ECH-associated protein 1 (*Keap1*) (192) enables nuclear translocation of nuclear factor erythroid 2-related factor 2 (*Nrf2*) (193, 194), which in turn engages antioxidant response elements and upregulates cytoprotective enzymes (193, 195). Prolonged hypobaric hypoxia, however, overwhelms systemic redox homeostasis, promoting excessive ROS accumulation (6, 58–60) and suppressing *Nrf2* expression along with its downstream antioxidant genes (191, 196), thereby crippling endogenous defense mechanisms.

ROS are generated through multiple interrelated pathways, including mitochondrial electron transport chain dysfunction (58, 197), NADPH oxidase activation (198), nitric oxide synthase uncoupling (197), and xanthine oxidase activity (199). Acute, intermittent, and chronic hypoxia each promote ROS buildup (6). When ROS production exceeds the scavenging capacity of endogenous antioxidant systems, oxidative stress ensues (200).

Excess ROS induce lipid peroxidation (59), particularly within polyunsaturated fatty acid-rich membranes, decreasing membrane fluidity (201, 202), increased permeability, and impairing membrane protein function (202). ROS also promote protein oxidation (60), and induce oxidative damage to both nuclear and mitochondrial DNA (199, 203). Collectively, these oxidative lesions across lipids, proteins, and nucleic acids constitute central mechanisms of irreversible neuronal injury in ischemic stroke (204).

Beyond direct cytotoxicity, oxidative stress amplifies ischemic brain injury by disrupting mitochondrial integrity (205), fueling inflammation (188, 191), triggering neuronal apoptosis (188), and compromising BBB integrity (188). Together, these pathways form a mechanistic bridge linking chronic hypoxic exposure to adverse cerebrovascular outcomes.

A summary of representative altitude- and duration-dependent adaptive and maladaptive responses is provided in Table 2.

## 6 Current limitations and future perspectives

### 6.1 Current limitations

Despite considerable advances, several important limitations warrant emphasis. A standardized framework for high-altitude exposure modeling remains lacking, particularly with respect to altitude gradients, exposure duration, and methods of hypoxic stimulation. This variability introduces substantial methodological heterogeneity across studies, complicating direct comparisons. Although accumulating evidence supports a transition from physiological acclimatization to maladaptive injury, the precise mechanisms governing this shift—and the interactions among distinct pathological pathways—remain incompletely understood. Moreover, much of the extant literature derives from controlled experimental hypoxia models, which may not fully recapitulate the complexity of long-term cerebrovascular adaptation and injury in real-world high-altitude environments. Collectively, these constraints limit mechanistic interpretation and reduce the translational generalizability of current findings.

### 6.2 Clinical implications and management challenges

Beyond unresolved mechanistic questions, several clinical salient challenges remain inadequately addressed in the context of high-altitude-associated ischemic stroke. Timely reperfusion therapy is paramount, as treatment delays are strongly correlated with poorer outcomes (206). In remote high altitude regions, logistical constraints—including limited healthcare infrastructure, suboptimal resource allocation, and inefficient workflows—can further delay definitive treatment (206, 207). Additionally, hypoxemia and excessive erythrocytosis, both characteristic of high-altitude physiology, may complicate clinical management and thrombotic risk stratification (208). Given the marked physiological heterogeneity among high-altitude populations, universally applicable management strategies remain undefined, and population-specific approaches are likely necessary (208). Prospective, high-quality studies are urgently needed to establish evidence-based clinical management strategies for ischemic stroke in these settings.

### 6.3 Future perspective

Future research should prioritize several key directions.

First, prospective studies that integrate clinical variables, advanced neuroimaging, circulating biomarkers, and machine learning-based analytics may refine risk stratification and facilitate individualized risk profiling for high-altitude-related ischemic stroke (209, 210). In alignment with this objective, our research group is conducting a multicenter prospective cohort study across the western Sichuan plateau (Chinese Clinical Trial Registry identifier: ChiCTR2400092762) aimed at developing prognostic prediction models based on clinical, neuroimaging, and multidimensional biological data (211).

Second, interventional studies should evaluate whether targeted therapies can sustain beneficial acclimatization while attenuate maladaptive responses during prolonged hypoxic exposure. Promising avenues include oxygen therapy (212, 213) and antioxidant strategies (214); however, their efficacy and optimal implementation in high-altitude-associated ischemic stroke remain to be rigorously established.

Third, altitude-specific prevention and management frameworks require systematic evaluation. Particular emphasis should be placed on clinically actionable domains, including blood pressure control, hemoglobin modulation, antithrombotic therapy, and equitable access to reperfusion treatment (215, 216). Current evidence remains insufficient to endorse standardized management protocols applicable across heterogeneous high-altitude populations. Well-designed prospective trials are therefore essential to define evidence-based preventive and therapeutic paradigms for ischemic stroke in high-altitude environments.

## 7 Conclusion

High-altitude exposure engages a biphasic response, operating along a continuum from physiological acclimatization to maladaptive injury. At mild-to-moderate elevations, coordinated compensatory responses preserve oxygen delivery and cerebrovascular homeostasis; with increasing altitude or prolonged exposure, however, maladaptive processes—including excessive erythrocytosis, hyperviscosity, endothelial dysfunction, blood–brain barrier disruption, neuroinflammation, and oxidative stress—elevate ischemic stroke risk (217). The balance between adaptation and maladaptation, shaped by altitude, exposure duration, and individual susceptibility, determines cerebrovascular outcomes in high-altitude populations. A refined understanding of this transition may inform precision prevention and management strategies in these geographically and physiologically distinct settings.
