# Correction: High-altitude exposure and ischemic stroke: pathophysiological mechanisms and current perspectives

**DOI:** 10.3389/fneur.2026.1945724

**Published:** 2026-08-06

**Authors:** Yuhuan Qiao, Yuding Luo, Yu Hu, Chuanxi Duan, Junhao Li, Xiaojing Luo, Jian Wang

**Affiliations:** 1 Department of Neurology, The Affiliated Hospital, Southwest Medical University, Luzhou, China; 2 Department of Neurology, Ya'an People's Hospital, Ya'an, China; 3 North Sichuan Medical College, Nanchong, China

**Keywords:** acclimatization, high altitude, hypoxia, inflammation, ischemic stroke, oxidative stress

Affiliation [National Institute for Stroke and Applied Neurosciences, Auckland University of Technology, Auckland, New Zealand] was erroneously included in the paper. This affiliation has now been removed for author(s) [Chuanxi Duan].

The conflict of interest statement has been correct to [The authors declare that the research was conducted in the absence of any commercial or financial relationships that could be construed as a potential conflict of interest.].

The original version of this article has been updated.

